# A comparative analysis of perceptual noise in lateral and depth motion: Evidence from eye tracking

**DOI:** 10.1167/jov.25.1.15

**Published:** 2025-01-24

**Authors:** Joan López-Moliner

**Affiliations:** 1 Vision and Control of Action (VISCA) Group, Department of Cognition, Development and Psychology of Education, Institut de Neurociències, Universitat de Barcelona, Barcelona, Catalonia, Spain

**Keywords:** continuous psychophysics, motion perception, perceptual noise, eye movements

## Abstract

The characterization of how precisely we perceive visual speed has traditionally relied on psychophysical judgments in discrimination tasks. Such tasks are often considered laborious and susceptible to biases, particularly without the involvement of highly trained participants. Additionally, thresholds for motion-in-depth perception are frequently reported as higher compared to lateral motion, a discrepancy that contrasts with everyday visuomotor tasks. In this research, we rely on a smooth pursuit model, based on a Kalman filter, to quantify speed observational uncertainties. This model allows us to distinguish between additive and multiplicative noise across three conditions of motion dynamics within a virtual reality setting: random walk, linear motion, and nonlinear motion, incorporating both lateral and depth motion components. We aim to assess tracking performance and perceptual uncertainties for lateral versus motion-in-depth. In alignment with prior research, our results indicate diminished performance for depth motion in the random walk condition, characterized by unpredictable positioning. However, when velocity information is available and facilitates predictions of future positions, perceptual uncertainties become more consistent between lateral and in-depth motion. This consistency is particularly noticeable within ranges where retinal speeds overlap between these two dimensions. Significantly, additive noise emerges as the primary source of uncertainty, largely exceeding multiplicative noise. This predominance of additive noise is consistent with computational accounts of visual motion. Our study challenges earlier beliefs of marked differences in processing lateral versus in-depth motions, suggesting similar levels of perceptual uncertainty and underscoring the significant role of additive noise.

## Introduction

Visual velocity is a cornerstone of daily function. This perceptual attribute allows us to update the changing position of moving objects (Whitney, 2002; Kwon, Tadin, & Knill, 2015; Aguilar-Lleyda, Tubau, & López-Moliner, 2018), giving us the ability to predict spatiotemporal properties of their trajectories like future positions (Finke, Freyd, & Shyi, 1986; Zago, Iosa, Maffei, & Lacquaniti, 2010; Diaz, Cooper, Rothkopf, & Hayhoe, 2013) or time to contact (Landwehr, Baurès, Oberfeld, & Hecht, 2013; Chang & Jazayeri, 2018; de la Malla & López-Moliner, 2022). Additionally, visual velocity informs the planning and execution of motor actions, such as the timing of interceptive movements (Marinovic, Plooy, & Arnold, 2012), where the motion of a target determines the temporal window for successful interception (Smeets & Brenner, 1995; Brouwer, Brenner, & Smeets, 2000). The necessity for precision in perceiving target motion is denoted by the complex sensorimotor coordination required in tasks like hitting a moving ball or navigating through traffic, where even small errors in velocity estimation can lead to significant real-world consequences.

The quest for quantifying precise visual speed perception has been central to psychophysical research. This goal has yielded numerous studies that have measured precision via comparative judgments, asking subjects to discern which of two stimuli is moving faster. Such research often uses psychometric functions to extract speed discrimination thresholds for various types of motion, including linear or fronto-parallel (De Bruyn & Orban, 1988; Watamaniuk & Duchon, 1992; de la Malla, Smeets, & Brenner, 2022), motion-in-depth (Harris & Watamaniuk, 1995; Portfors-Yeomans & Regan, 1996; Brooks & Mather, 2000; Rushton & Duke, 2009), and more complex movements like rotation or spiral motion (Clifford, Beardsley, & Vaina, 1999). A recurring finding across these studies is the higher thresholds detected for motion-in-depth compared to fronto-parallel motion (e.g., Rushton & Duke, 2009; Aguado & López-Moliner, 2019), hinting at a fundamental sensitivity difference. This can be contrasted with the quite good performance humans exhibit in visually-guided real-world tasks, such as catching (López-Moliner & Brenner, 2016), even with accelerated objects (Brenner et al., 2016), denoting a discrepancy between poor temporal precision measured by judgements and practical expertise. Arguably, timing interceptive actions can rely on time-to-contact rather than speed in real-life performance and this higher-order variable can be obtained by combining optical variables (e.g., angular size and its rate of expansion), leading sometimes to smaller thresholds than those obtained for the rate of expansion (Regan & Hamstra, 1993). However, evidence also suggest that people would need to recover target speed accurately to adjust the speed of final movement adjustments (Brouwer et al., 2000; López-Moliner & Keil, 2012). The proficiency observed in real-world interactions with motion-in-depth suggests that there may be additional factors at play beyond the basic sensory thresholds measured with classical psychophysical methods. One possibility is that the reliance on conscious access to velocity information in judgment-based tasks may lead to an overestimation of speed thresholds. Conscious perception of speed is often subject to individual variability and is perceived as a challenging task, which could inflate the reported thresholds. Furthermore, the exceptionally low thresholds sometimes reported for fronto-parallel motion are often based on data from a limited number of highly psychophysically trained participants. For instance, De Bruyn and Orban (1988) report Weber fractions between 5% to 10% for speeds from 2 to 64°/s. In contrast, de la Malla et al. (2022) find Weber fractions between 15% and 30% for speeds spanning 1.3°/s to 20°/s, figures which could be more indicative of the uncertainty within the general population.

One method of measuring speed precision without relying on psychophysical judgments involves analyzing smooth pursuit eye movements and their accuracy in adjusting to changes in target velocity. This approach allows for the derivation of oculometric functions, which can be compared directly to psychometric curves. Some previous studies (e.g., Kowler & McKee, 1987; Gegenfurtner, Xing, Scott, & Hawken, 2003) have reported a striking similar accuracy and precision between psychometric and oculometric thresholds. This could be initially regarded as pursuit and speed perception sharing the same sources of noise, which would result in trial-by-trial correlations between error in pursuit and psychophysical judgements as shown by Stone and Krauzlis (2003). However, this correlation has not always been found despite the similarity of psychometric and oculometric curves (Gegenfurtner et al., 2003). More recent evidence (Tavassoli & Ringach, 2010) suggests that performance of the oculomotor system faithfully reflects sensitivity to motion signals, which is consistent with the initial capturing of pursuit direction by the perceived direction of a moving stimulus (Montagnini, Mamassian, Perrinet, Castet, & Masson, 2007). Whether the same noise limits pursuit and perception is still an open debate (see Spering & Montagnini, 2011 for a review) and is in close relation with which is the contribution of motor noise in pursuit. This aspect is especially relevant for the present study because we aim at measuring perceptual uncertainty with pursuit, and it is presented in some detail.

### Sensory and motor noise

The discussion around the contributions of sensory versus motor noise in pursuit is particularly concentrated on the initial or open loop phase of smooth pursuit, that is mainly before the feedback including both retinal and extraretinal signals arrive. Osborne, Lisberger, and Bialek (2005) propose that sensory noise is the main source of variability of eye movements during this phase. They found that more than the 90% of the pursuit variability could be accounted for by consequences of errors of sensory estimates. However, this conclusion was later challenged by Rasche and Gegenfurtner (2009). This study reported a substantial initial contribution of motor noise that diminishes over time, eventually becoming minimal by the steady-state phase.

During the closed loop phase, the perceptual and motor components become highly intermingled, as evidenced by research indicating that a re-afferent copy of the eye movement command is used in the computation of perceived speed (Freeman & Banks, 1998; Turano & Heidenreich, 1999; Turano & Massof, 2001). This complexity makes it more difficult to distinguish between the two types of noise. Our approach assumes that the relative contribution of motor noise during the closed-loop phase, which we use to estimate perceptual noise, is smaller than that of sensory noise and, crucially, remains consistent across different conditions.

Additionally, it is important to consider that the influence of motor noise in eye pursuit may be less significant compared to other types of movement, such as hand movements. Studies such as the one by Bonnen, Burge, Yates, Pillow, and Cormack (2015) have observed larger thresholds in continuous psychophysical uncertainties for hand movements compared to two-alternative forced choice (2AFC) judgments, suggesting that motor noise may play a more pronounced role in tasks requiring fine motor control. This distinction underlines the need to contextualize motor noise contributions relative to the specific movement being analyzed.

### Additive and multiplicative noise

In light of these considerations, our research aims to measure speed uncertainty from a continuous psychophysics approach (Bonnen et al., 2015); relying on models of smooth eye pursuit (e.g., Orban de Xivry, Coppe, Blohm, & Lefevre, 2013) to estimate speed sensory uncertainty from retinal slip. Smooth pursuit eye movements are closely tied to the visual system's ability to perceive and predict target motion, offering a direct measure of visual motion processing with higher temporal precision and less reliance on conscious judgment (Huk, Bonnen, & He, 2018), making it highly useful for measuring speed.

We aim to estimate speed uncertainty for lateral and in-depth motions during smooth pursuit tasks. Although previous studies using continuous psychophysics also have focused on lateral motion and motion-in-depth, they have used random walk dynamics (Bonnen et al., 2017), without the predictive motion component based on speed (Kwon et al., 2015; Aguilar-Lleyda et al., 2018). Our work builds on prior research (Décima, Barraza, & López-Moliner, 2022), which applied a Kalman filter in estimating relative speed uncertainty in contrast-induced motion bias through manual tracking.

One potential additional advantage of using a smooth pursuit model is the consideration of both additive and multiplicative noise in the sensory processing. *Additive noise* refers to variability in sensory processing (or motor responses) that is not influenced by the magnitude of the signal. In contrast, multiplicative noise scales with the signal, implying that as the speed or intensity of a stimulus increases, so does the variability in the response. In the context of motor control, multiplicative noise is known as signal-dependent noise and is operational for explaining the endpoint variability in the execution of motor actions like reaching or pointing (Harris & Wolpert, 1998). This noise is key to explain saccade supression (Crevecoeur & Kording, 2017) following optimality principles. Although these two different kind of noise are discussed under psychophysical theory (e.g., Kingdom & Prins, 2016), deriving the associated noise from classical discrimination tasks is not straightforward and needs further assumptions about the shape of the transducer because both types of noise could be compatible with a given performance under the assumption of different transducer functions (Zhou, Duong, & Simoncelli, 2024). Continuous psychophysics can then offer a viable solution to disentangle the noise associated with visual speed and this particular aspect has been integrated in inverse optimal control theory applied to continuous psychophysics (Schultheis, Straub, & Rothkopf, 2021). Specifically in the study of eye pursuit, signal-dependent noise suggests that the precision of eye movements is not static but fluctuates in accordance with the characteristics of the target's motion. This aspect is integrated in an initial sensory phase of a model of pursuit (Orban de Xivry et al., 2013) as a Kalman filter to compute perceptual uncertainty during smooth pursuit eye movements. This approach would allow us to quantify the additive and multiplicative noise inherent in the pursuit system, providing a more complete picture of the uncertainties involved in perceiving visual speed. By characterizing these noise components, we can see how they contribute to previously reported velocity thresholds. We will do this using immersive displays to quantify speed uncertainty through a tracking task and determine how thresholds for lateral motion and motion-in-depth differ when velocity is an inherent aspect of the motion. This approach also expands the methodological repertoire of continuous psychophysics by leveraging a virtual reality environment for the computation of perceptual uncertainties through motor responses, providing a novel perspective on speed perception.

## Methods

### Participants

The study involved the same group of 10 participants, all of whom participated in Experiments 1 (random walk) and 3 (nonlinear motion). For Experiment 2 (linear motion), eight participants were included, three of whom also participated in Experiments 1 and 3. Participants had an age range of 27 to 35, with a gender distribution of four self-identified as women and the rest as men. All participants had normal or corrected-to-normal vision and reported no history of neurological disorders.

The study was part of a program that was approved by the Ethical Board of the University of Barcelona (Institutional Review Board IRB00003099), conforming to the standards of the Declaration of Helsinki. All participants signed an informed consent before taking part in the study.

### Apparatus

The experiment was conducted using an Intel i7-based PC (Intel, Santa Clara, CA, USA). The visual stimuli were generated by an NVIDIA GeForce GTX 1070 graphics card and displayed on an HTC Vive Pro head-mounted display (HMD) at 90 Hz per eye, with a resolution of 2880 × 1600. The HMD provides a field of view of 100° horizontally and 110° vertically for each eye. The participant's head position in three-dimensional (3D) space (x, y, and z coordinates) and angular orientation (yaw, pitch, and roll) were recorded by two SteamVR BASE STATION 2.0 at a frequency of 90 Hz. Eye movements, specifically the combined gaze angle, were tracked using the HMD's built-in eye-tracking system, which operates at a sampling rate of 120 Hz with an accuracy of 1.08 degrees and a standard deviation precision of 0.36 degrees (Schuetz & Fiehler, 2022). On the temporal side, the eye tracker has a known latency of 50 ms (Stein et al., 2021), which is significant when interpreting correlograms between stimuli and pursuit in the random walk condition. However, this delay does not impact the estimation of uncertainty with the Kalman filter, because the stimulus and response time series will be aligned in time, as in Bonnen et al. (2015). Because we aim at estimating precision with smooth pursuit, we wanted to know the amount of noise that the eye tracking equipment added to the recordings. Similarly to Rasche and Gegenfurtner (2009), we used a model eye (Eyelink; SR Research, Ottawa, Ontario, Canada) and recorded the eye tracking data while the model eye was totally still. We identified a specific frequency of noise (0.71 Hz) in the built-in eye tracker recording that was removed by applying a Butterworth filter. Participants underwent a standard calibration procedure before each session, and the interocular distance between the two displays of the HMD was individually adjusted.

### Experimental design and stimuli

**Figure 1.**
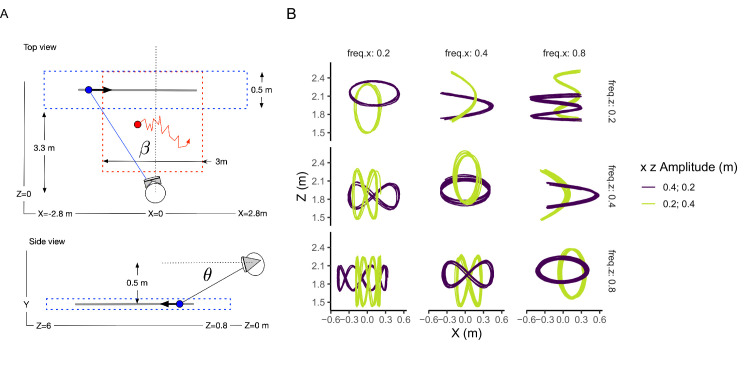
Experimental stimuli and setup. (**A**) Geometry of the trajectories used in the experimental conditions: random walk and linear motion. Top view: The blue dotted rectangle shows the area in which lateral (fronto-parallel) linear trajectories could be shown. The gray line shows an example of trajectory with a target (blue dot). The gaze angle ***β*** is defined between the target and the line x = 0 in world coordinates. The red dotted square (3 m × 3 m) show the area in which the random walk trajectories could be shown. Side view: The blue rectangle show the span of the trajectories in-depth. The gaze angle ***θ*** is defined between the target and the line crossing the eye height parallel to the ground. (**B**) The trajectories used in the nonlinear motion (Lissajous paths) for the different lateral (column-wise panels) and in-depth (row-wise panels) temporal frequencies. The color codes the two possible amplitudes.

Participants were exposed to three different motion conditions (see Figure 1) in a virtual environment generated with Unity software. They were instructed to follow a moving target (a white sphere of 5 cm diameter) against a black background. In all conditions the height of the sagittal plane containing the target movement was 0.5 m below eye height. The three conditions were as follow:

• **Random walk movement (Condition 1):** The target motion was defined by a two-dimensional random walk process in the sagittal plane (x, z). The position *x* and *z* of the target at time t + 1 was determined by the following equation:
  $$\begin{array}{c} x_{t+1}=x_{t}+\nu_{x};\;\;\nu_{x}∼N\left(0,\sigma \right) \end{array}$$
  $$\begin{array}{c} z_{t+1}=z_{t}+\nu_{z};\;\;\nu_{z}∼N\left(0,\sigma \right) \end{array}$$
  where ν_*x*_ and ν_*z*_ are independently normally distributed random variables with a standard deviation σ of 0.01 m. This noise represents the process noise (true stimulus noise) which will be essential for accurately estimating the positional measurement noise reported later by using a Kalman filter with a random walk dynamics (Bonnen et al., 2015). The motivation of the this condition is to allow a more direct comparison of our results with those reported by Bonnen, Huk, and Cormack (2017) in a 3D tracking task and see whether estimates with two different experimental settings (VR environment and a more traditional display system) are comparable. It will also provide a baseline for understanding how the visual system processes motion when the movement of the target is unpredictable. As reported in Bonnen et al. (2017), we expect to find a worse performance for motion-in-depth. By including this condition, we can also have a better understanding of how linear and nonlinear predictive components (next conditions) help update position information.
• **Linear motion (Condition 2):** The target moved with a constant speed in a straight line. Each trial had one of the following physical speeds: 0.47, 0.61, 1.00, 1.65, or 2.12 m/s. These values in metric units corresponded to angular speeds between 6°/s to 28°/s in the lateral motion condition and between 0.66°/s and 17°/s in the in-depth direction. The physical speed values are in the following geometrical progression: 1.0  ×  exp(−0.75,   −0.5,  0,  0.5,  0.75) with a geometric mean of 1.0. Assuming Weber's law in the perception of speed, this distribution would be more appropriate because of increases in the difference between consecutive speeds for larger values of the signal. The target traveled distance had a mean of 2.22 m (SD = ± 0.05 m), so the duration ranged from 1.05 sec for the maximum speed to 4.73 sec for the slowest one. The direction of the motion was constant during the trial, but varied, moving either in the frontal-lateral (x) or depth (z) direction between trials. To introduce process noise, a random positional noise with a standard deviation of 0.005 m was added to the target's position at each time step. The purpose of this noise will be evident when introducing the Kalman filter below, but now it is important to mention that an experimenter can only approximate the internal or process noise of the Kalman filter, later introduced as *Q*, with the actual variance of the stimulus. This approach follows the methodology used by Bonnen et al. (2015) and Falconbridge, Stamps, Edwards (2023), where the actual variance of the stimulus noise is utilized in data analysis to infer the observation variances.
• **Nonlinear motion (Lissajous curves, Condition 3):** In this condition, the target's motion followed complex, looping patterns known as Lissajous curves (see Figure 1B). These curves result from combining two sinusoidal motions in orthogonal directions (x and z). The movement in the x and z directions was defined by sinusoidal functions with specific frequencies and amplitudes, creating Lissajous patterns. Frequencies used for the sinusoidal components were 0.2, 0.4, or 0.8 Hz, and amplitudes were set at 0.2 or 0.4 m. These parameters combined, resulting in a variety of Lissajous curves with distinct shapes and complexities (see Figure 1B). Similar to Condition 2 (linear motion), a random positional noise with a standard deviation of 0.005 m was introduced to the target's position at each time step, adding process noise to the system.

### Procedure

Participants were standing and wore the VR headset. The eye-tracker calibration was performed at the start of each session. Participants were instructed to follow the white sphere with their eyes as accurately as possible throughout the experimental session.

In the random walk condition, participants were shown a total of 68 trials in two different blocks. Each trial lasted 21 seconds.

In the linear condition participants were shown with 10 repetitions for each combination of the five values of physical speed, direction (lateral, depth) in each of the two blocks of Trials. They were shown then a total of 200 trials.

In the nonlinear movement condition participants were shown with 36 Trials per block (3 lateral frequencies * 3 in-depth frequencies * 2 amplitudes [x = 0.2 and z = 0.4 or x = 0.4 and z = 0.2] * 2 repetitions). They participated in three blocks, making a total of 108 trials.

All the participants took part in the random walk condition first. The order of the other two conditions was counterbalanced across participants.

### Data analysis

In our study, the motion of a target within a three-dimensional space (specifically in the x, z axes, with y held constant) was simulated using metric units in the immersive space to accurately represent physical motion. However, for the purposes of analysis, including the Kalman filter fitting and the computation of cross-correlation functions, we converted these motion measurements into retinal coordinates (angular units) based on the observer head position and orientation to have target in the same frame of reference as the gaze. The angles β and θ shown in Figure 1 illustrate respectively the angular position in z (depth) relative to the observer and the lateral position. This conversion was essential for calculating variables like pursuit gain and retinal slip. This methodological conversion from physical to angular units introduced a distinction in the retinal speed range for lateral versus motion-in-depth conditions. Although the metric speeds remained consistent across conditions, their angular unit equivalents diverged, leading to a broader range of angular speeds in the depth condition compared to lateral movements. Despite these disparities in speed ranges when expressed in angular units, there was sufficient overlap between the conditions to allow for meaningful comparisons.

#### Cross-correlograms in the random-walk condition

Each trial in all conditions provided a pair of x-z (lateral-depth) time series. The y position was fixed and consequently was not included in the analysis. We computed the first temporal derivative of target and combined gaze position to obtain the respective target speed and gaze velocities for each trial and separately for the lateral and depth component of motion. The time series based on target and gaze speed were used to compute the cross-correlogram (CCG) for each trial for the lateral and depth components. We computed the CCG for each participant and condition individually. For this analysis, we skipped the first 4 sec of motion. We fitted a skewed Gabor function (Bonnen et al., 2017) to each individual CCG by minimizing the least squared error defined by the following piece-wise function of time *f*(*t*):

$$\begin{array}{ccc} & & \;f\left(t\right)=\left\{\begin{array}{cc} a\cdot \exp\left(-\frac{{\left(t-\mu \right)}^{2}}{2\cdot \sigma_{2}^{2}}\right)\cdot \sin\left(2\pi w(t-\mu )\right) & \mathrm{for}\;\;\;t<\mu \\ a\cdot \exp\left(-\frac{{\left(t-\mu \right)}^{2}}{2\cdot \sigma_{1}^{2}}\right)\cdot \sin\left(2\pi w(t-\mu )\right) & \mathrm{for}\;\;\;t\geq \mu \end{array}\right. \end{array}$$

This piece-wise function is a sine functions convolved with a skewed Gaussian as a function of time lag t; *a* is the amplitude, µ is the mean, and σ_1_ and σ_2_ are the SD for the two Gaussians defined respectively above and below the mean. Finally ω denotes the frequency of the sinus. We fitted these five parameters using the *optim* function in the R software R Development Core Team (2007). We compute, from the fits, the time at which the above function is maximum (lag), as well as the maximum value of the function (peak). These two parameters denote the processing delay and accuracy in the tracking response.

Note that the CCG analysis can only provide an interpretable pattern for open-loop conditions that do not incorporate feedback, such as the random-walk condition in our study. However, we apply this analysis in Condition 2 as well to obtain a relative processing delay measure between the two motion directions.

#### Oculometric functions in the linear motion condition

We use the method developed by Gegenfurtner et al. (2003) to obtain measures of precision for lateral and in-depth motion directions. This analysis will provide another set of comparisons of precision between the two direction conditions, which will serve to validate the sensory uncertainties obtained with the Kalman filter presented below. The oculometric functions were built from receiver operator characteristic (ROC) analysis of pursuit velocity distributions for the different five speeds and participants in the linear motion condition. The ROC curves fitted to each speed condition allow us to compute a proportion of faster than the median speed value (in our experiment 1 m/s). This proportion is obtained by computing the area and the ROC curve. We finally fitted cumulative Gaussian functions to these proportions and obtained a measure of discriminability (i.e., the SD of the Gaussian) and comparable Weber fractions between the two motion conditions. We used quickpsy software (Linares & López-Moliner, 2016) to fit the oculometric functions by maximizing the log-likelihood. In addition to the mean an SD of the cumulative Gaussian we also fitted guesses and lapses, that is lower and upper asymptotic values, respectively.

### Estimating sensory uncertainty

In this study, we adopt the framework of the retinal-motion model (Brenner & van den Berg, 1994; Turano & Heidenreich, 1999) to explicitly relate uncertainty in retinal slip (RS) to uncertainty in perceived velocity. Retinal slip (*RS*) represents the difference between the retinal image motion and the compensatory eye velocity ($\dot{E}$) during pursuit eye movements, expressed as: $RS=\dot{V}-\dot{E}$, where $\dot{V}$ is the physical velocity of the moving object. This relationship denotes that RS provides direct access to $\dot{V}$ together with an extra-retinal visual motion signal (Turano & Massof, 2001). Therefore we assume that variations in RS directly reflect fluctuations in the perceived velocity of objects in the visual field.

#### Kalman filter model for noise estimation

We used a Kalman filter model, based on the model of eye pursuit movement by Orban de Xivry et al. (2013), which uses the retinal slip as input, to estimate additive and multiplicative observation noise from the eye-tracking data. The main dependent variable in our analysis was the actual retinal slip ($RS_{k}^{act}$), defined as the difference between target speed and current eye speed, or the image velocity at time (*k*).

##### Process equation and noise observation

The sensory processing of retinal slip was modeled using a process equation with Gaussian noise:

$$\begin{array}{c} RS_{k+1}^{Sens}=RS_{k}^{Sens}+\theta_{k},\;\;\;\;\;\;\theta_{k}∼N\left(0,Q\right) \end{array}$$

where *Q* represents the SD of the process noise. The brain will register noisy observations of retinal slip ($RS_{k}^{Obs}$) that were modeled as

$$\begin{array}{ccc} & & \;RS_{k}^{Obs}=RS_{k}^{Sens}+\gamma_{k}RS_{k}^{Sens}+\nu_{k},\\ & & \;\gamma_{k}∼N\left(0,D\right);\;\;\nu_{k}∼N\left(0,R\right) \end{array}$$

with γ_*k*_ and ν_*k*_ representing multiplicative and additive measurement noises with respective SD of *D* and *R*. These two measurement uncertainties are the objective parameters we aim to estimate.

##### Kalman gain and update equation

The Kalman filter estimated the noise parameters D (multiplicative noise) and R (additive noise) using the update equations:

$$\begin{array}{ccc} & & \;{\hat{RS}}_{k+1}^{Sens}={\hat{RS}}_{k}^{Sens}\\ & & \;+\;K_{k}\left(RS_{k}^{Obs}-{\hat{RS}}_{k}^{Sens}\right)+\eta_{k},\;\;\eta_{k}∼N\left(0,\Omega \right) \end{array}$$

Here, η_*k*_ represents internal noise of the Kalman estimate with variance Ω^2^, held constant (Ω = 0.3) based on previous estimates Orban de Xivry et al. (2013). The Kalman gain (*K_k_*) was computed assuming Gaussian and uncorrelated noise (Todorov 2005) as

$$\begin{array}{ccc} & & \;K_{t}=\Sigma_{k}^{Sens}\\ & & \;{\left(\Sigma_{k}^{Sens}+R^{2}+D^{2}\left(\Sigma_{k}^{Sens}+{\hat{RS}}_{k}^{Sens}{\left(\hat{RS}k^{Sens}\right)}^{T}\right){\left(D^{2}\right)}^{T}\right)}^{-1}, \end{array}$$

and the error covariance ( $\Sigma_{k}^{Sens}$ ) was updated as:

$$\begin{array}{c} \Sigma_{k+1}^{Sens}=Q^{2}+\Omega_{k}^{2}+\left(1-K_{k}\right)\Sigma_{k}^{Sens} \end{array}$$

### Data fitting and parameter estimation

The recorded retinal slip using the eye tracker data was considered as the perceptual response and consequently as the posterior estimate ${\hat{RS}}_{k}^{Sens}$. We minimized the root mean square (RMS) error between the estimated sensory retinal slip ${\hat{RS}}_{k}^{Sens}$ and the actual tracking data to fine-tune the parameters *R* and *D*. Unlike the traditional use of the Kalman filter where the observation noise is known, we use the Kalman to estimate the observational uncertainties *R* and *D*. Accurate estimates of these uncertainties depend on knowing the process variance *Q*^2^. As experimenters, we do not have access to this process internal variance, but we can approximate this internal variance with the actual variance of the stimuli (*Q*^2^), as in Bonnen et al. (2015) and Falconbridge et al. (2023). This is based on the assumption that participant's estimates of the noise in the stimuli will be closed to the actual ones. Since we introduced positional noise only experimentally, we need to compute the true velocity noise (*Q*) in the stimuli to accurately estimate *R* and *D*. We extracted the velocity data by calculating the first temporal derivative of position, and the standard deviation of the velocity changes provided an empirical estimate of *Q*. The average value of *Q* was 0.04°/s.

## Results

### Data statement

To detect outliers, for each trial we computed the average difference between target and gaze. We removed all the trials in which this difference was larger than 2°. In the random walk condition, the average percentage of outliers was 8.6%, which was due to one participant with 63% outliers; after removing this participant the percentage of outliers was 4.7%. In the linear motion condition, the mean percentage of outliers was 9.6 %, which was caused by one single participant having 37% of outliers. This participant was removed from further analysis, resulting in an average number of trials detected as outliers of 5.8%. In the nonlinear condition the initial percentage of outliers was 14%, which was caused, again, by a single participant. Once this participant was removed, the percentage of outliers was 4.9%.

### Tracking analysis

#### Random walk

**Figure 2.**
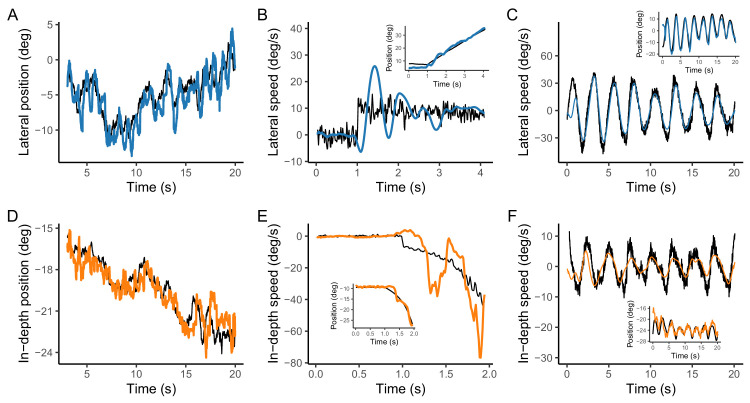
Trajectory examples. Examples of individual trials for the lateral (top panels) and in-depth (bottom panels) components of motion. The different conditions are shown column-wise with the target motion (position or speed) in black and gaze in blue (lateral) or orange (in-depth). (**A**) lateral position of the target and gaze in the random walk condition; (**B**) Target velocity and gaze velocity in the linear motion. (**C**) Target speed and gaze speed in the Lissajous paths. Panels **D**, **E**, and **F** show the same information or the in-depth component. The insets in panels **B**, **C**, **E**, and **F**, which show trajectory speeds, plot the corresponding target and gaze position versus time.

**Figure 3.**
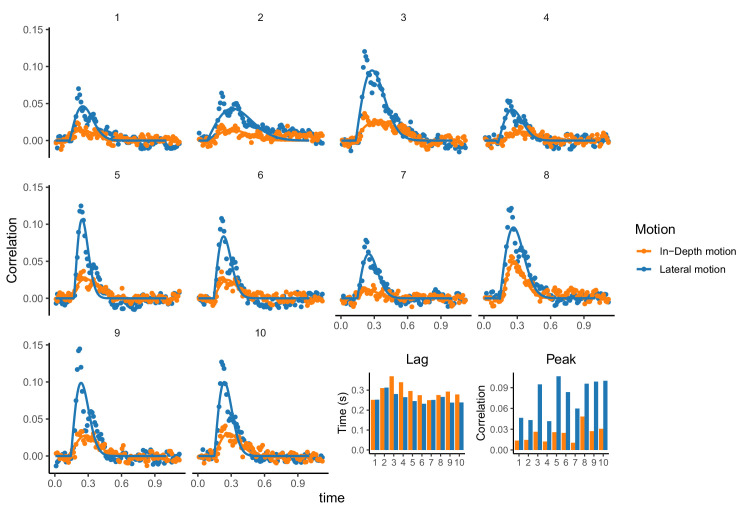
Cross-correlogram analysis. Cross correlograms for each participant (numbered panels from 1 to 10) in the random walk condition for the two motion components (lateral in blue and depth in orange). The lines denote the best fit of the skewed Gabor function. The lag (time of maximum correlation) and peak (value of the correlation) are shown for each participant on the bottom right part of the figure.

Figures 2A and 2D show two representative examples of pursuit trajectories for lateral (A) and depth (D) motion components. As can be seen, participants could track the target motion in the two dimensions without difficulties. However, tracking performance in-depth was impaired with respect to tracking of the lateral motion component as shown by the individual CCGs and the corresponding parameters (see Figure 3). The estimated lag value for the in-depth component was significantly larger (longer delay in tracking) than the lag for the lateral component (*t*(9) = −3.4233, *p* = 0.0075) and the peak (correlation) was significantly smaller than the lateral (*t*(9) = 8.914, *p* < 0.001). We also found a significant difference in the width of the CCGs between lateral and depth (not shown) with the in-depth component having larger width of the Gabor functions (*t*(9) = −2.85, *p* = 0.019) This pattern is consistent with previous work (Bonnen et al., 2017) that shows an impairment of tracking for the depth component in a random walk motion dynamics.

Part of the observed difference between lateral and in-depth motion can be attributed to the difference in their projected retinal step sizes. Our condition aligns closely with the initial experiment of Bonnen et al. (2017). In their investigation, Bonnen et al. introduced a specific condition designed to normalize size compression effects associated with motion in depth. Despite this control, they noted that differences persisted between the two dimensions, albeit to a lesser extent. Because our findings are in close agreement with their work using a different display system, we think that controlling for retinal size compression could mitigate some disparities in perceived motion between lateral and depth directions, but it would not entirely eliminate them.

#### Linear motion

Figures 2B and 2E show respectively a lateral (B) and an in-depth (E) trials of two physical linear trajectories. The nonlinearity in the in-depth speed is caused by the projection of the trajectory onto the retinal plane (see Figure 1A). One can notice the noise (*Q*) added to the stimulus that is treated as a process noise in the Kalman filter model presented above. In both cases it is conspicuous an spontaneous oscillatory pattern in the pursuit speed that has been reported often in previous studies (Robinson, 1965; Fuchs, 1967; Robinson, Gordon, & Gordon, 1986; Goldreich, Krauzlis, & Lisberger, 1992), and that can be accounted for by an underdamped oculomotor plant. Often, we see a speed overshoot in the initial part of the pursuit; however, this overshoot was not included when estimating the oculometric functions and the uncertainty estimates.

The average pursuit gain was not affected by the motion direction: 0.85 and 0.89 for the lateral and in-depth trajectories respectively (*F*(1, 63) = 0.98; *p* = 0.32). Neither target physical speed (*F*(4, 63) = 2.4; *p* = 0.06) nor the interaction of velocity and motion direction (*F*(4, 63) = 0.43; *p* = 0.77) were significant. Interestingly, when comparing the lag obtained from the CCG, the processing delay between the two motion directions (lateral vs. motion-in-depth) was not significantly different (*F*(1, 69) = 2.43, *p* = 0.123).

**Figure 4.**
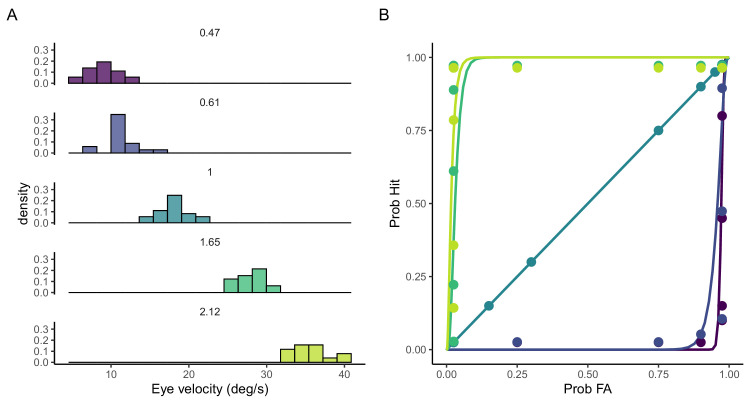
ROC analysis. (**A**) Distribution of pursuits peeds for the different physical velocities for one participant and motion direction (lateral). (**B**) ROC curves computed from the distributions in (**A**) taking asa base speed the mid speed (1 m/s) and for 15 criterion. The area under the ROC curves gives the proportion of faster than the base speed.

To measure pursuit performance in a way that is comparable to perceptual performance, we obtain the ROC curves from the distributions of eye velocity for each participant, stimulus physical speed, and motion direction. Figure 4A illustrates the eye speed distributions for one participant under one condition of motion direction. The underlying idea is that the more separated these distributions, the higher the sensitivity. Figure 4B shows the corresponding ROC curves obtained by applying 15 criteria on the eye speed distributions.

**Figure 5.**
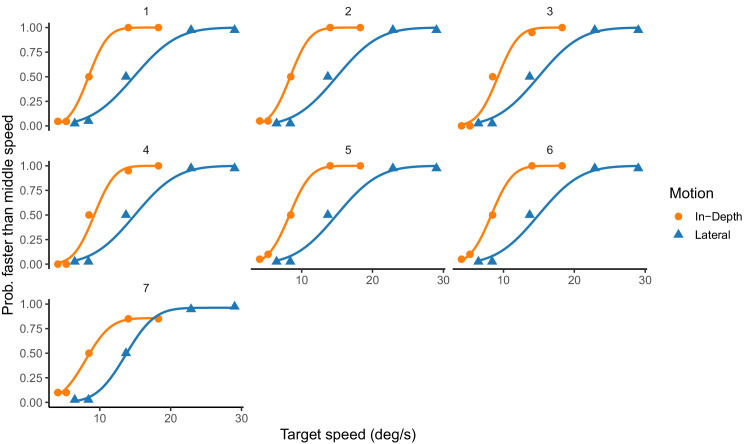
Oculometric functions. Oculometric functions for all participants and motion direction. The areas under the ROC curve in Figure 4 allow us to reconstruct the oculometric functions the slope of these functions denote the sensitivity or discrimination threshold. The curves stand for the best fit of a cumulative Gaussian function.

From the area under the different ROC curves that would represent proportion of faster than the base speed responses we built the oculometric functions for each participant and motion direction. Figure 5 shows the oculometric functions and the best fit of a cumulative Gaussian curve.

To compare the performance between the two motion directions we obtained the Weber fractions for each participant and motion direction based on the slope (parameter SD of the cumulative Gaussian). The obtained Weber fractions ranged from 0.28 and 0.34 for the lateral condition and were between 0.28 and 0.37 for the motion in-depth. The Weber fractions were not statistically different between the two condition (paired *t*-test, *t*(6) = 1.12, *p* = 0.30). The Weber fractions obtained for each participant and motion condition, ranging between 0.27 and 0.35, are relatively large. One plausible explanation for this is that the speeds used in the experiment may have been too widely spaced relative to the participants’ perceptual thresholds. The curves for most participants saturated at the two extreme speeds, indicating that these speeds were easily distinguishable from the mid one, but the wide physical separation between them likely contributed to larger discrimination thresholds.

#### Nonlinear motion

Figures 2C and 2F show an example for the lateral (C) and in-depth (F) motion components. Note that, unlike in the linear motion condition where the two components were shown in different trials, here the two components are part of the same coherent motion with a smooth transition from one to the other.

**Figure 6.**
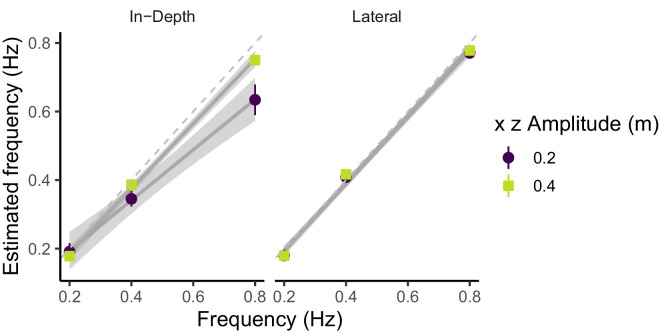
Frequency analysis. Estimated temporal frequency of the pursuit movements versus the temporal frequency of the stimuli. The color codes the amplitude of the stimulus displacement. The different motion components (lateral and in-depth) are shown in different panels.

To obtain an idea of the quality of the tracking for each temporal frequency, we estimated the frequency of the pursuit movement for each motion component by computing the FFT. Figure 6 plots this estimated frequency as a function of the stimulus frequency and split per movement amplitude or displacement. As can be seen, only in the depth movement and smaller amplitude the pursuit does not reflect the frequency of the stimuli, specially at the highest frequency (slope smaller than 1: 0.74 [0.58–0.87]).

Although the pursuit gain was larger in the lateral motion (0.89) than in the in-depth motion (0.79), the difference did not reach significance (*F*(1, 54) = 1.36; *p* = 0.25). Only the temporal frequency of the motion affected the pursuit gain (*F*(2, 54) = 6.1; *p* = 0.004) with lower pursuit gains with higher frequencies. The interaction was not significant.

### Position and velocity uncertainty

#### Random walk

**Figure 7.**
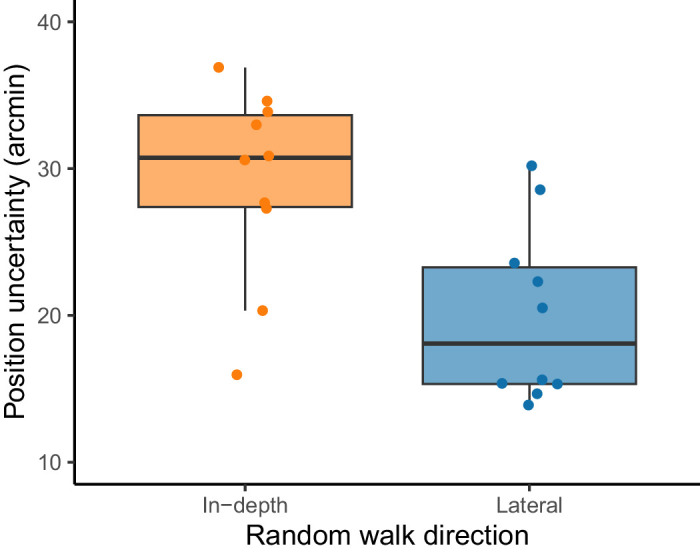
Position uncertainty for the random walk condition. The dots denote the individual observational uncertainties or measurement noise as estimated from a Kalman filter. The horizontal jittering is for the sake of presentation.

In the random walk condition, positional uncertainty was estimated using a Kalman filter, following the same approach of Bonnen et al. (2015). We independently estimated the positional observational (measurement) noise for each participant in both motion directions (lateral and in-depth). The estimated observational noise, representing positional uncertainty, is presented in Figure 7. These uncertainty values are comparable to those reported in Bonnen et al. (2015) and were significantly smaller for the lateral condition compared to the in-depth condition (*t*(9) = 3.11, *p* = 0.0125, paired *t*-test). This pattern is consistent with the larger lag and smaller peaks observed in the cross-correlograms for the in-depth direction, as shown in Figure 3.

#### Linear motion

**Figure 8.**
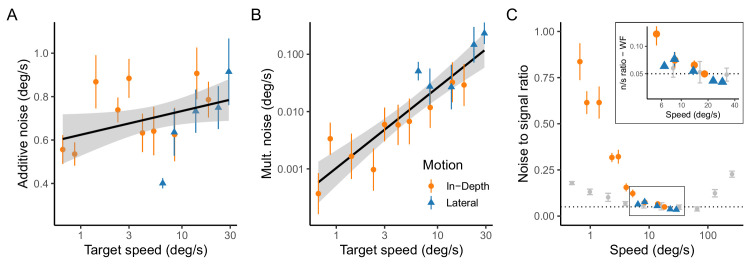
Visual uncertainty for uniform motion. (**A**) Estimation of additional noise (as standard deviation SD) component of the Kalman filter (R) as a function of target speed for the two motion directions: lateral and in-depth. (**B**) Estimation of multiplicative noise (D) for the two directions of motion expressed as SD. (**C**) Noise to signal ratio based on the total noise (multiplicative + additive). The inset zooms in the area of the rectangle for the sake of comparison. In all panels, direction of motion is coded by color and shape. The black lines in panels A and B denote the best linear fit. The gray dots in panel C denote the Weber fractions obtained in De Bruyn and Orban (1988). Error bars denote 95% CI.

Figure 8 shows the main results of the estimated velocity uncertainty obtained from the Kalman filter. The additive and multiplicative noise that affect the observations of retinal slip are plotted in Figures 8A and 8B, respectively. The additive noise in tracking the linearly moving target at constant speed (expressed in SD) shows a flat trend across motion directions. This suggests, as expected, that additive noise remains relatively consistent across a range of speeds with values between 0.5° and 1°/s. This indicates that the internal noise added to the system is not significantly influenced by the speed of the target, which is an important consideration in computational models of visual motion (e.g., Stocker and Simoncelli, 2006). We will resume this point in the discussion. Panel B of Figure 8 shows the multiplicative noise (also expressed as SD multiplied by the signal), which scales with the speed of the target. This type of noise is proportional to the signal itself, implying that as the target moves faster, the uncertainty in tracking its speed increases. The linear trend in the plot, along with its log-scale, indicates a power-law relationship between speed and multiplicative noise. The fit has a slope of 0.96 (95% CI = 0.68–1.25) which implies actually a linear relation between the speed signal and the multiplicative noise.

Panel C in Figure 8 shows the noise to signal ratio as a normalized measure of sensitivity to changes in stimulus speed. This noise to signal ratio can be regarded as an approximation of the Weber fraction and is calculated based on the ratio of the total noise (combining additive and multiplicative noise) to the speed of the target. The main plot shows the common initial decreasing trend, suggesting that the observer's sensitivity to speed changes improves at higher speeds until it reaches a minimum in our data. The gray dots represent the Weber fractions (based on 84% discrimination thresholds) reported in a previous study (De Bruyn & Orban, 1988) that measured a larger range of speeds. However, for similar tested speed values, shown within the squared and the inset (zoomed-in version), these Weber fractions are quite similar to our noise to signal ratio, including the both lateral and depth components. However, direct comparisons between these two uncertainties should be approached with caution, as the uncertainty measured by the Kalman model represents the noise for a single frame, without accounting for temporal integration. This point is revisited in the discussion.

The observed uncertainty pattern, indicated by orange for in-depth and blue for lateral motion across all panels, implies that motion direction influences observation noise. In-depth motion consistently exhibits elevated levels of both additive and multiplicative noise, resulting in higher noise to signal ratio relative to lateral motion. This trend may be indicative of the visual system's differential processing mechanisms for depth perception, which are likely more complex due to the additional extra-retinal information required for accurate depth discrimination (e.g., Brenner, van den Berg, & van Damme, 1996; Murdison, Leclercq, Lefèvre, & Blohm, 2019). Nonetheless, at comparable speed values, the uncertainty profiles for in-depth and lateral motion converge, suggesting a larger degree of uniformity in the visual system's handling of speed information when motion directions are matched for retinal velocity.

The actual retinal speeds are well below the upper limit at which pursuit is impaired (about 90°/s; Meyer, Lasker, & Robinson, 1985). As introduced above, the pursuit gain as an additional indicator of the quality of the pursuit was not different for lateral and in-depth motion. This suggest that the quality of the pursuit did not affect the estimated uncertainty.

#### Nonlinear motion

**Figure 9.**
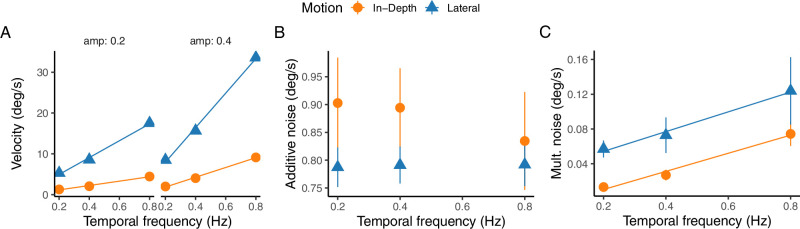
Observation uncertainty for the nonlinear motion. (**A**) Average retinal speed as a function of the temporal frequency. The two panels show the data for the two movement amplitudes. (**B**) Estimated additive noise from the Kalman filter against temporal frequency for the two motion directions (color and symbol coded). (**C**) The same as panel B for the multiplicative noise.

Figure 9 presents the uncertainty estimates for non-linear motion tracking, specifically of Lissajous paths as illustrated in Figure 1B. These paths are unique in that the amplitude and temporal frequency directly influence both the physical and retinal velocities of the motion.

Panel A of Figure 9 plots the relationship between velocity and temporal frequency for the two movement amplitudes (0.2 and 0.4 m), respectively. The data reveal a pronounced difference in the rate at which retinal velocity increases with temporal frequency—a difference more pronounced in lateral (blue triangles) than in in-depth (orange circles) motion. This disparity stems from the motion's retinal projection, and should be kept in mind for interpreting the subsequent perceptual noise results in Figures 9B and 9C.

Figure 9B illustrates the estimated additive noise versus temporal frequency, showing a tendency for in-depth motion to have higher noise levels across frequencies. However, this difference was not statistically significant (*F*(1, 54) = 1.26, *p* = 0.26), nor were the effects of temporal frequency (*F*(2, 45) = 0.91; *p* = 0.4097) or their interaction (*F*(2, 45) = 1.74, *p* = 0.187). Yet, when considering the average retinal speeds, which are lower for in-depth motion compared to lateral motion for identical frequencies, the signal-to-noise ratio is notably reduced for in-depth motion (*F*(1, 45) = 41.7; *p* < 0.0001). The spread of additive noise values aligns with those obtained from the linear motion condition (compare with Figure 8A).

Multiplicative noise, as plotted in Panel Figure 9C, demonstrates a significant increase with frequency (*F*(2, 54) = 5.04, *p* = 0.009) and is higher for lateral motion (*F*(1, 54) = 13.31, *p* < 0.001), with no significant interaction (*F*(2, 54) = 1.30, *p* = 0.28). The range of multiplicative noise is consistent with that derived from linear motion (see Figure 8C), underscoring the robustness of these noise estimates across different motion types. In sum, Figure 9 showcases that despite the complexity introduced by nonlinear Lissajous paths, the perceptual noise characteristics remain comparably stable to those observed in linear motion, affirming the reliability of these measures in diverse motion conditions.

## Discussion

In this study we have estimated speed perceptual uncertainties based on a Kalman filter of a pursuit model (Orban de Xivry et al., 2013) for lateral and in-depth directions of motion within an immersive environment. In agreement with previous work (Bonnen et al., 2017), we have replicated an inferior performance for motion-in-depth when the target position dynamics followed a random walk. However, our results show that the reported lower sensitivity for discriminating speed of motion-in-depth (e.g., Rushton & Duke, 2009; Aguado & López-Moliner, 2019) should be considered cautiously.

### Lateral versus motion-in-depth: A predictive component matters

The observed distinction between lateral and motion-in-depth performance is significantly influenced by the ability to predict future positions based on velocity (Kwon et al., 2015). We have observed that the disparity between lateral and motion-in-depth is significantly amplified in conditions where the motion dynamics follow a random walk, rendering future positions unpredictable. We can reproduce then previous published results that compared the two directions (Bonnen et al., 2017). In their study the tracking in the depth dimension was impaired yielding smaller peaks (correlation coefficients in the CCGs), larger lags or delays, and larger widths of the fitted Gabors functions to the CCGs. Our results in the random walk condition are in total agreement.

When future positions can be anticipated based on velocity, the difference between lateral and motion-in-depth estimates of uncertainty is less marked. One of the reasons for this is related to our experimental stimuli. In our case, motion-in-depth occurred within the sagittal plane, positioned 0.5 meters below eye height, diverging from the conventional approach of employing isotropic expansion/contraction to investigate motion-in-depth precision (Portfors-Yeomans & Regan, 1996; Rushton & Duke, 2009) or time-to-contact estimation (López-Moliner & Bonnet, 2002; Hosking & Crassini, 2011; López-Moliner, Supèr, & Keil, 2013). Isotropic expansion suggests an object is on a collision course or directly approaching the observer, and it has been a predominant model in previous studies focusing on motion-in-depth to evaluate perceptual thresholds or time to contact. However, isotropic expansion represents a specific case of motion-in-depth. Although this scenario is valuable for studying certain aspects of depth perception, such as timing interceptive actions or estimating time to contact, it may not adequately represent situations where objects move within the same plane as the observer but at varying distances, such as the conditions simulated in our study. Our choice was partly motivated by the complexities associated with tracking a target under isotropic expansion, which can be challenging because of the inherent difficulty in maintaining a stable focus on expanding or contracting stimuli without a clear lateral or vertical reference point.

This finding further supports the notion that velocity plays a predictive role in anticipating changes in the target's depth position. Notably, within the range of retinal speeds for which we obtained measurements for both motion directions, there was virtually no difference in the estimated observation noise, as highlighted in the inset of Figure 8. The similarity between the relative measured noise represented by the noise to signal ratio and previously established Weber fractions from speed discrimination tasks (De Bruyn & Orban, 1988) is very high. This observation is at odds with earlier reports of higher measurement noise in tracking tasks compared to judgment tasks (Bonnen et al., 2015), which required the application of more comprehensive models to separate motor noise from perceptual noise (Straub & Rothkopf, 2022). We resume this point later.

In addition to our primary uncertainty analyses, we derived oculometric functions to further explore the differences (or lack thereof) between lateral and in-depth motion conditions. We did so for the uniform motion condition in which we could apply the ROC analysis to obtain the oculometric functions. Our results failed to find significant differences in the oculometric functions between these two motion directions, in agreement with the similar observation noise when the lateral and motion-in-depth speeds were comparable. However, when comparing the Weber fractions derived from these oculometric analyses to the uncertainties estimated using the Kalman filter, we observed that the Weber fractions were consistently larger. One possible explanation for this is that the speeds used in the experiment may have been too widely spaced resulting in response saturation at the two extreme speeds. Consequently, the wide physical separation between these speeds likely contributed to larger discrimination thresholds. Another caveat when interpreting the oculometric curves is that the slopes for the lateral condition might have been overestimated compared to the in-depth motion because of the larger spacing between speeds in the lateral condition. Although we did not find significant differences between the two conditions in the oculometric analysis, the validity of this result remains uncertain and should be interpreted with caution.

Importantly, the observational noise estimated using the Kalman filter corresponds to single-frame uncertainties. This factor has also been identified as a potential source of discrepancy between the observational uncertainties estimated from the tracking task (Kalman) and the positional thresholds derived from the 2AFC task (Bonnen et al., 2015). The difference likely arises because temporal integration plays a larger role in the 2AFC task. Temporal integration may also influence the estimation of speed uncertainty in tracking tasks. In our current Kalman model, we do not explicitly account for the temporal integration factor, which complicates direct comparisons between uncertainties estimated with classical psychophysical methods. Addressing this limitation would require the design of new experimental tracking paradigms and the incorporation of temporal integration into the models. Such efforts would help clarify the relationship between Kalman-derived estimates and thresholds (or Weber fractions) obtained through classical psychophysics. It is important to recognize that continuous psychophysics remains a relatively new and evolving field. Extending existing models—or developing new ones—to account for temporal integration and other perceptual processes, both for position and speed, will be essential for advancing this area of research.

### Additive and multiplicative noise

Utilizing the pursuit model to estimate perceptual uncertainties from retinal slip (Orban de Xivry et al., 2013) offers the distinct advantage of distinguishing between additive and multiplicative noise. While classical psychophysical approaches also allow for this distinction, they rely on various assumptions regarding the transducer function and noise characteristics (e.g., Kingdom & Prins, 2016; Zhou et al., 2024). In our research, the estimation of these two types of noise across two distinct conditions (Figures 8 and 9) denotes their anticipated behaviors: additive noise remains relatively constant across different speeds, whereas multiplicative noise escalates with the speed's magnitude. Notably, the additive noise estimated in both conditions significantly surpasses the multiplicative noise. This finding aligns with computational models of visual motion processing that predominantly propose additive noise as the principal noise type (Simoncelli, Adelson, & Heeger, 1991). However, signal-dependent noise is implied in more recent models of speed perception (Stocker & Simoncelli, 2006) where the estimated width of the likelihood is constant width in the log-speed domain.

Despite the smaller magnitude of the multiplicative noise, one could also wonder why this type of noise is needed in the linear motion condition, where the physical speed is constant. The presence of multiplicative noise in this condition could be attributed to the following causes. Even though the physical speed of the stimulus is uniform, the retinal speed from which we compute the retinal slip, is not. This is the case, especially when the target is moving in-depth. In addition to this, as shown in Figure 2, gaze velocity shows typical oscillatory patterns reported elsewhere (Robinson, 1965; Fuchs, 1967; Robinson et al., 1986; Goldreich et al., 1992). These nonlinearities would also affect the measured retinal slip leading to an increase in multiplicative noise as these fluctuations may be signal-dependent as considered in the motor control literature (Harris & Wolpert, 1998; Crevecoeur & Kording, 2017; Schultheis et al., 2021).

### Assumed linearity of perceived speed

The pursuit model we employed assumes a linear relationship between pursuit (eye) speed and perceived speed, implying the existence of an extraretinal linear transducer that maps eye speed to perceived speed. In reality, perceived speed likely involves the combination of two transducers: one for retinal speed and another for extra-retinal signals (Freeman, 2001). The precise shapes of these transducers remain under debate. Nevertheless, the assumption of linearity in our model is supported by previous evidence showing that a parameterized linear model effectively captures the relationship between extraretinal speed and perceived speed in the absence of retinal motion, as demonstrated under the zero base velocity condition in Turano and Massof (2001).

Discrimination tasks from classical psychophysics face significant challenges in inferring the transducer's shape (Kingdom, 2016). This is because discrimination performance can align with multiple transducer shapes, depending on whether the underlying noise is assumed to be multiplicative or additive Zhou et al. (2024). Alternative approaches, particularly in the context of contrast perception, suggest combining discrimination and scaling experiments (Kingdom, 2016), or more recently, using magnitude estimation (Zhou et al., 2024) as a direct method for inferring the transducer.

The continuous psychophysics framework, which flips the conventional use of the Kalman filter (Bonnen et al., 2015), offers additional tools for estimating both perceptual noise and internal response states—key components of internal representations. To date, the main frameworks assume a linear mapping between physical stimuli (true states) and internal responses, combined with additive Gaussian noise Straub and Rothkopf (2022). Although methods to account for signal-dependent noise on the motor side are available (Schultheis et al., 2021), approaches to estimate nonlinear perceptual mappings and uncertainties in continuous tasks remain an open area for future development. In the meantime, accurately measuring uncertainty may need to focus on visual features where a linear relationship between physical stimuli (state) and perceptual response can be reasonably assumed. Perceived speed from pursuit eye movements appears to be one such case.

### Speed and potential confounds

A critical consideration in motion perception research is the potential confound introduced by variables correlated with speed, such as duration or displacement. In experiments relying on speed judgments (e.g., comparing which of two stimuli appears faster), participants might base their judgments on this correlated variable, potentially affecting the thresholds. However, Continuous Psychophysics methodology diverges from traditional judgment-based approaches. By deriving perceptual noise through a tracking task, we can circumvent the direct influence of duration or displacement as a confounding variable. The congruence observed between the uncertainty measures in our study and those reported in classical studies, which meticulously controls for these confounds (as shown in the inset of Figure 6C), underscores the benefits of this methodology. This alignment affirms the efficacy of Continuous Psychophysics in accurately capturing perceptual uncertainties without the interference of potential biases.

However, this is a different aspect than considering also position as a relevant visual variable for smooth pursuit. Like in a previous study (Décima et al., 2022), we chose to use retinal slip, and therefore a velocity-based only model, to estimate speed uncertainty. This is motivated by most of smooth pursuit models relying on retinal speed only (Gegenfurtner et al., 2003; Lisberger, 2010). However perceived target position has been proposed (Pola & Wyatt, 1980) as an important control variable for smooth pursuit. Enhanced models that include position and its integration with velocity could be used in future research to estimate conjointly both uncertainties.

### The role of motor noise

Previous studies have observed increased uncertainties in tracking tasks compared to those derived from judgment-based tasks for position information (Bonnen et al., 2015). This discrepancy has led to the development of more comprehensive frameworks, such as those incorporating Linear Quadratic Gaussian (LQG) principles (Straub & Rothkopf, 2022), which extend beyond the ideal observer model represented by the Kalman filter. The LQG framework is particularly advantageous as it can account for both proprioceptive and motor noise, offering a more nuanced understanding of the sources of uncertainty in motor tasks. The contribution of early (perceptual) versus late (motor) noise in tracking different perceptual attributes has also been explored by simpler models based on ideal observer analysis (Ambrosi, Burr, & Cicchini, 2022). This model suggest that the primary source of noise occurs early in visual processing, indicating that suboptimal response efficiency is due to perceptual noise rather than random motor variability.

In our study, we did not conduct experiments based on speed judgments per se. Instead, we compared our findings on speed uncertainties with those from prior research and found no significant overestimation for the similar speed values. This observation could suggest that motor noise might have played a lesser role in our experimental conditions. One possible reason for this is the predictive nature of our velocity stimuli. Unlike random walk stimuli, where future positions are unpredictable, the linear and non-linear motions we employed allowed participants to anticipate the target's trajectory, thereby reducing the need for continuous motor adjustments or planning. This reduced reliance on constant motor planning and execution (van Beers, 2009) in response to newly perceived positions might have minimized the influence of motor noise on our findings. This assumption is consistent with previous findings from smooth pursuit that point to a lesser impact of motor noise in the closed-loop or steady phase of pursuit (Osborne et al., 2005; Rasche & Gegenfurtner, 2009).

Importantly, we assume that motor noise is similar in both motion directions—lateral and in-depth. Given that our task involved comparable motor demands in these two directions, it is reasonable to expect that the motor noise would be consistent across conditions. This assumption is supported by the lack of significant differences of the oculometric functions, the processing lag and the pursuit gain between the two motion directions, suggesting that the precision of eye movements was not disproportionately affected by the direction of motion.

Additionally, it is important to note that existing models in the literature that consider motor noise, including those based on LQG principles, typically assume that motor noise is additive, Gaussian, and the dynamics are linear (Straub & Rothkopf, 2022), or are more suited for hand movements (Falconbridge et al., 2023). These factors limit the direct application of these models to our experiment. Future research could address this by extending the speed range in lateral motion conditions, allowing for more direct comparisons with classical methods and providing further insights into the nature of motor noise in different motion contexts.

## Conclusions

Leveraging the higher temporal resolution offered by continuous psychophysics methods (Huk et al., 2018), our study not only reveals that the perceptual uncertainties -referred to as measurement noise- between lateral motion and motion-in-depth might not be as distinct as previously believed but also allows us to show that the perceptual noise in processing visual speed is predominantly additive. This methodology enables us to bypass potential limitations introduced by conscious access (i.e., judgements) to these attributes, which could affect the accuracy of measured thresholds.
